# Omentopexy with Glubran®2 for reducing complications after laparoscopic sleeve gastrectomy: results of a randomized controlled study

**DOI:** 10.1186/s12893-019-0507-7

**Published:** 2019-11-05

**Authors:** Vincenzo Pilone, Salvatore Tramontano, Michele Renzulli, Mafalda Romano, Angela Monda, Alice Albanese, Mirto Foletto

**Affiliations:** 1 General, Bariatric and Emergency Surgery Unit of Fucito Hospital, University Hospital of Salerno, Salerno, Italy; 2 Week Surgery Unit, University Hospital of Padova, Padova, Italy

**Keywords:** Sleeve gastrectomy, Omentopexy, Sealant, Leak, Gastric fistula

## Abstract

**Background:**

Gastric fistulas, bleeding, and strictures are commonly reported after laparoscopic sleeve gastrectomy (LSG), that increase morbidity and hospital stay and may put the patient’s life at risk. We report our prospective evaluation of application of synthetic sealant, a modified cyanoacrylate (Glubran®2), on suture rime, associated with omentopexy, to identify results on LSG-related complications.

**Methods:**

Patients were enrolled for LSG by two Bariatric Centers, with high-level activity volume. Intraoperative recorded parameters were: operative time, estimated intraoperative bleeding, conversion rate. We prospectively evaluated the presence of early complications after LSG during the follow up period. Overall complications were analyzed. Perioperative data and weight loss were also evaluated. A control group was identified for the study.

**Results:**

Group A (treated with omentopexy with Glubran®2) included 96 cases. Control group included 90 consecutive patients. There were no differences among group in terms of age, sex and Body Mass Index (BMI). No patient was lost to follow-up for both groups. Overall complication rate was significantly reduced in Group A. Mean operative time and estimated bleeding did not differ from control group. We observed three postoperative leaks in Group B, while no case in Group A (not statistical significancy). We did not observe any mortality, neither reoperation. Weight loss of the cohort was similar among groups. In our series, no leaks occurred applying omentopexy with Glubran®2.

**Conclusion:**

Our experience of omentopexy with a modified cyanoacrylate sealant may lead to a standardized and reproducible approach that can be safeguard for long LSG-suture rime.

**Trial registration:**

Retrospective registration on clinicaltrials.gov PRS, with TRN NCT03833232 (14/02/2019).

## Background

Bariatric surgery is currently considered a stable and safe solution for morbid obesity. Different surgical options are available, and they are continuously evolving, influenced by new reports of literature [1, 2]. Real question is today comprehension, prevention and ideal treatment of major complications, reducing morbidity and mortality [3]. In particular, laparoscopic sleeve gastrectomy (LSG) is actually the most performed bariatric procedure in most countries and since 2009 the American Society for Metabolic and Bariatric Surgery (ASMBS) estabnilished LSG as distinct bariatric procedure [4]. Moreover, a number of serious, sometimes fatal, complications must be considered, like gastric fistulas or suture line dehiscence (leaks), bleeding, and strictures [5, 6]. Leaks/gastric fistulae, although appearing in a low percentage of patients, increase morbidity and hospital stay and may put the patient’s life at risk. Many risk factors and preventive technical details, in order to reduce this event, have been proposed, with discordant results [6–8]. We report our prospective evaluation of application of synthetic sealant, a modified cyanoacrylate [N-Butil-Cyanoacrylate (NBCA) + Metacrylosysolfolane (MS), a co-monomer owned by GEM S.r.l. – Viareggio (LU) –Italy], defined Glubran® 2, on suture rime, associated with omentopexy, to identify results on LSG-related complications.

## Methods

The prospective randomized trial is designed with the aim to verify the effectiveness of the Glubran®2 used in its spray application, according to manufacturer’s indications, to perform the omentopexy of the staple line to prevent and reduce early complications after LSG. single-blind randomization was explained: a single surgeon, in enrollment phase, assigned patient to case or control group, after adequate communication of randomization to all patients. The surgeon that performed procedure only knew if patient was randomized to case group (LSG with omentopexy with Glubran®2) or to control group (LSG without omentopexy with Glubran®2). Control group was identified for the study with simple randomization, considering patients treated with LSG during same period. Patients of case and control groups were not paired. Same recording was performed for both groups. Patients were enrolled for LSG by two Bariatric Centers, with high-level activity volume, after multidisciplinary evaluation: inclusion criteria, according with international guidelines [1, 9], was body mass index (BMI) of greater than 40 kg/m^2^ or > 35 with at least one co-morbidity, such as hypertension, dyslipidemia or diabetes, age ≥ 18 years old, medically unfit for surgical intervention, absence of active gastric disease, of uncontrolled medical or psychiatric conditions, and signed written informed consent. Bariatric procedure was performed according with standardized four-trocars technique [10]. All surgeons involved had a proved experience for bariatric surgery, and have completed learning curve.

The size of the boogie to be used for calibration ranged from 42 to 48 Fr, among two groups. In case group, after gastric partition and confirming correct closure of mechanical section (performed with Endo-Gia, varying depth of stapler, from blue to green charge, according with gastric level), we applied a layer of the synthetic sealant on all rime suture and chose an omentum flap to place and cover it. We carefully controlled absence of gastric rotation with omentum flap, or any tension on the resected stomach. In control group, we reinforced staple line with buttressing (bovine pericardium) of mechanical stapler, or with running suture of the rime alone, indifferently. A recording of type of reinforcing was performed, also if not pertinent to study.

Anthropometric data recorded were: age, weight, BMI, presence of comorbidities. Intraoperative recorded parameters were: operative time, estimated intraoperative bleeding (in ml), conversion rate. We prospectively evaluated the presence of early complications after LSG during the follow up period (30 days from intervention). Considered complications were staple line leakage/gastric fistula, postoperative bleeding, intraabdominal abscess, cardiopulmonary failure, and all other complications. In order to considering effects and real impact of mentioned events, we also evaluated length of hospital stay, rate of readmission, rate of reintervention, overall mortality at 30 days. Weight loss was recorded at 15 and 30 days, as excess weight loss percent (EWL%) and as reduction of BMI.

The continuous variables were presented as mean ± standard deviation. The demographic data and perioperative data were compared using the student’s t and Mann- Whitney U tests for continuous variables, while Fisher’s exact test was used to determine any statistical significance for the categorical variables. The level of significance was set at 0.05.

All procedures involving human participants were in accordance with the 1964 Helsinki declaration and its later amendments.

## Results

Enrollment of case and control group was performed between January and April 2017. Case group (treated with omentopexy with Glubran®2) included 96 cases. All patients were enrolled for bariatric surgery and LSG was for all indicated (Table 1). Control group included 90 patients treated with LSG without omentopexy with Glubran®2. In all cases, laparoscopic procedure was performed according with standardized technique.

**Table 1:** anthropometric data of Group A and B

	Group A	Group B
Number	96	90
Male/Female	44/52	32/58
Age (mean; years)	37.4 ± 3.5	39.6 ± 5.0
Weight (kg)	118.4 ± 13.9	126.5 ± 14.2
BMI (kg/m^2^)	44.6 ± 4.1	45.7 ± 3.8
Supeobese pts. (BMI > 50; number, %)	20 (20.8%)	22 (24.4%)
Comorbidities (%)	64.6 (62 cases)*	71.1 (64 cases)*
Hypertension	51	58
OSAS	1	1.2
CD	10	7.5
Dyslipidemia	47.5	40
Diabetes	21.8	30

*p: 0.09

*BMI* body mass index

*OSAS* obstrtuctive sleep apnea syndrome, *CD* coronaric disease

There were no differences among group in terms of age, sex and BMI. No patient was lost to follow-up for both groups.

Intraoperative data and follow-up are explained in Table 2: overall complication rate was significantly reduced in case group. In 68.8% (62 cases) of control group buttressing of bovine pericardium was applied, while in other cases (31.2%; 28 cases) suturing was preferred. Mean operative time and estimated bleeding did not differ from control group. All cases of postoperative bleeding, recorded in case and control groups, were solved with blood transfusion and conservative therapy. We did not observe any postoperative leak in case group, until the 30-days follow-up, while three leaks in control group, recovered on 3th and 4th postoperative day, were recorded (p: 0.08). All were treated with conservative approach and supportive medical therapy, until to radiologic disappearance. Number of patients does not enhance to any statistical significance. No difference of complication rate was observed for two types of reinforcing (suturing or buttressing), in control group (data not shown).

**Table 2:** intraoperative data and follow-up

	Group A	Group B
Mean operative time (minutes)**	83.3	75.3
Estimated IOBL (ml)**	100	150
Overall complications (%)*	3.1 (3 cases)	20 (18 cases)
Postoperative bleeding**	2.1 (2 cases)	4.4 (4 cases)
Fistula/Gastric leak***	0	3.3 (3 cases)
Intraabdominal ascess***	0	4.4 (4 cases)
Cardiopulmonary failure****	1.0 (1 case)	2.2 (2 cases)
Wound infection****	0	1.1 (1 case)
Other complications****	0	4.4 (4 cases)
Hospital stay (days)***	4.5 ± 1.5	5.8 ± 2.0
Mean drain removal time (PO day)**	4.2 ± 0.5	5.3 ± 0.6
Readmission rate (%)*****	2.0	1.8
Reintervention rate (%)	0	0
Overall mortality at 30 days	0	0

*IOBL* intraoperative blood loss

*p < 0.05

** p: 0.08

***p: 0.07

****p: 0.09

*****p: 0.06

**Fig. 1:**
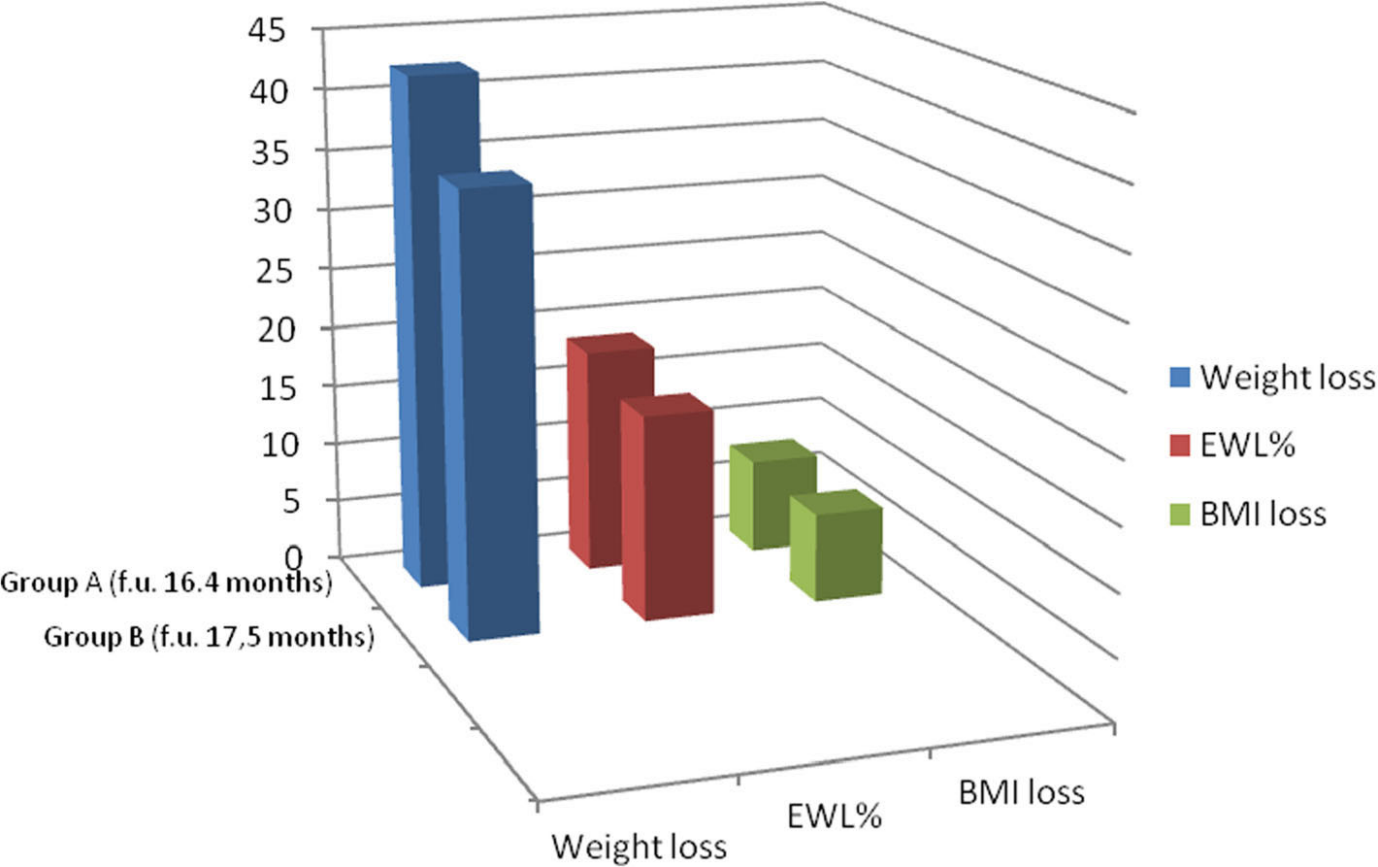
weight loss related to follow-up

We did not observe any mortality, neither reoperation, at a mean follow-up of 16.4 months for Group A and of 17.5 months, for Group B. Regarding to postoperative data, mean drain removal time, mean hospitalization and reintervention rate did not significantly differ from control group (Table 2). Weight loss of the cohort, synthetized in Fig. 1, was similar among groups.

## Discussion

Laparoscopic sleeve gastrectomy (LSG) is a standardized bariatric procedure in all Occidental countries. Its mechanism of action include early satiety and gastrointestinal hormonal variation, including reduction of ghrelin levels [4, 5]. In relation to dramatic results on weight loss, also on long-term follow-up, LSG is recently overcoming on all major bariatric procedure, as single procedure, with a minority of cases in which a second malabsorptive procedure should be proposed [3, 4]. Moreover, LSG is associated with high-risk complications, that determine also a prolonged hospitalization, increasing home care, and significant mortality risk [5].

Based on the data of more than 12.000 LSGs, the International Sleeve Gastrectomy Expert Panel Consensus Statement 2011 showed the leak rate was 1.06% [11], while overall complication rate is near to 15% [12]. From the revision of the literature the leak rate can vary between 1 and 3% for primary procedure, with an overall leak associated mortality of 9% [6] [12, 13].

Considering pathogenesis of this complication, early leaks within 48 h are caused by a technical defect: stapler misfire, or wrong staple size for the tissue, are possible factors [14]. Late leaks, occurring after several days, are surely related to tissue ischemia caused by tension on the anastomosis, distal bowel obstruction, or hematoma. In both situations, the intraluminal pressure is demonstrated and determinant for fistulization [14].

In a multicenter experience with 2834 patients, leaks post LSG were related to abnormal vascularization, bleeding or thermal injuries. Demonstrated risk factors were increased age, male, gender, sleep apnea and revisional surgery. Actually, one of the most discussed topic is the best prevetion of this complication [15].

Various preventive techniques have been proposed. The use of closed suction drain routinely near the staple line, despite that it is performed by the majority of surgeons, may not be helpful for diagnosis [16]. The size of the boogie to be used for calibration is also a subject of controversies, ranging between 32 and 60 Fr: a large systematic review taking 4888 patients and another large meta-analysis of 9991 patients suggested that larger boogie size may decrease the leak rate, but statistical significance for boogie size on leak rate is lacking [17]. Staple line reinforcement has commonly diffused, in order to reducing weak points of suture and bleeding risk, with different products on stapler or putting after resection, such as fibrin sealants. These were investigated in majority of studies, with good impact in term also of decreasing leakage rate, on the rationale the polymerization process also acts as a sealant to prevent leaks [6, 18]. All large randomized prospective trials and meta-analysis showed no significant difference between reinforcement (by oversewing or buttress on stapler) and simple section, in term of leakage rate [19, 20]. On the other hand, most authors agree that reinforcement decreases bleeding risks. On this line, Gagner et al., in a powerful review on 88 papers, shows in the no reinforcement group an overall complication rate of 8.9%, and confirmed that buttressing LSG suture did not determine a statistical evidence on leaks [21]. Almost all device making for reinforcing determine generally a faster procedure compared with oversewing suture, that is suitable for experienced surgeons [22].

Conversely, omentopexy has been historically evaluated to reinforce perforated peptic ulcer, or after bronchial dehiscence. Local application on gastrointestinal anastomosis has been reported: in all cases an omentum flap is located on sutured tract with reinforcing stitches [23, 24]. The only bariatric report on omentopexy reported a possible effect on gastrointestinal symptoms, after LSG, without results on mitigating food discomfort [25]. Recently, some other authors believe that in duodenal switch omentopexy over lateral gastric staple line and around as much of the gastrostomy to buttress it together drainage and feeding jejunostomy could be efficacious to prevent ischemic leaks [26]. Moreover, the effect of the staple line omentopexy using a sealant synthetic glue despite the sutures to prevent postsurgical LSG complications has never been investigated before. Cyanoacrylate sealant seems to be comparable to fibrin glue in staple line reinforcement, in recent comparative analysis [27]. We hypothesize that NBCA+MS sealant (Glubran®2) may add a major action on staple for bleeding, and, either fixing omentum either enhancing adhesive action, reduce risk of leak. It has proved its adhesive effect, confirming as excellent sealant and hemostatic agent. All these properties are necessary to guarantee an effective buttressing of the staple line.

Our comparative prospective evaluation seems indicate that omentopexy with NBCA+MSsealant is a safe and reproducible technique, with good results on bleeding and leaks. We found a significant results on overall complications, despite no difference was observed for specific ones, on single statistical evaluations. In detail, leak rate was higher for control group, although without significance, enhancing value of sealant and omentopexy for reinforcing suture rime.

Although significant results are not evident, operative time is not conditioned, neither weight loss. Operative time is comparable to classic LSG; similarly technical aspect of omentopexy is not determinant, neither it determined a more complex procedure. Conversely, double effect, haemostatic and on tension suture, seems to be guaranteed. It has discussed the possible adhesive effect of omentopexy, in case of bariatric second-time: in this case, enlargement of sleeve is a key to perform with good safety a gastrointestinal anastomosis and/or a new gastric section, also including minimal residual sealant in the excluded stomach. Increase of adherence risk is not clearly demonstrated for all hemostatic agent. Larger studies are mandatory, to confirm our new observation, that, differently from numerous trial about fibrin glue and oversewing techniques, can be easily standardized and is more physiological for action of omentum. In fact, rate of employed glue is very poor compared to fibrin products.

## Conclusion

LSG is a safe technique, but staple line-associated complications can be life-threatening. In this series, no leaks occurred applying omentopexy with NBCA+MS glue (Glubran®2), from the very beginning of the surgeons’ experience in LSG. Actually, there is no conclusive evidence to suggest that routine oversewing of the staple line or reinforcement with buttressing material after LSG decreases these complications. Proper mentoring, and performance of surgery in appropriate settings are good approaches to decreasing complications. Our experience of omentopexy with amodified cyanoacrylate sealant may lead to a standardized and reproducible approach that can be safeguard for long LSG-suture rime.

## Abbreviations

ASMBS: American Society for Metabolic and Bariatric Surgery
EWL%: Excess weight loss percent
LSG: Laparoscopic sleeve gastrectomy
MS: Metacrylosysolfolane
NBCA: N-Butil-Cyanoacrylate
